# Quantifying Stern layer water alignment before and during the oxygen evolution reaction

**DOI:** 10.1126/sciadv.ado8536

**Published:** 2025-03-05

**Authors:** Raiden Speelman, Ezra J. Marker, Franz M. Geiger

**Affiliations:** Department of Chemistry, Northwestern University, 2145 Sheridan Road, Evanston, IL 60660, USA.

## Abstract

While water’s oxygen is the electron source in the industrially important oxygen evolution reaction, the strong absorber problem clouds our view of how the Stern layer water molecules orient themselves in response to applied potentials. Here, we report nonlinear optical measurements on nickel electrodes held at pH 13 indicating a disorder-to-order transition in the Stern layer water molecules before the onset of Faradaic current. A full water monolayer (1.1 × 10^15^ centimeter^−2^) aligns with oxygen atoms pointing toward the electrode at +0.8 volt and the associated work is 80 kilojoule per mole. Our experiments identify water flipping energetics as a target for understanding overpotentials, advance molecular electrochemistry, provide benchmarks for electrical double layer models, and serve as a diagnostic tool for understanding electrocatalysis.

## Teaser

Water flipping occurs before the oxygen evolution reaction starts.

## INTRODUCTION

Much microscopic insight into the Stern layer water structure and the electric fields at electrolyte:electrode interfaces currently comes from atomistic simulations (*1*–*7*), with joint theoretical and surface-specific experimental studies just emerging (*8*–*12*). Probing interfacial solvent structure and electrostatic fields at electrode:electrolyte interfaces directly, in real time, and without the need for electrochemical, spin, or spectroscopic labels, plasmonic structures, or arbitrarily chosen reference states remains a major challenge despite the topic’s importance for many electrochemical transformations (*7*, *13*–*18*). The major challenge is water’s strong absorber problem, complicating the detection of water’s stretching and bending modes at electrode:electrolyte interfaces. Compounding the problem is that linear spectroscopies are insensitive to whether water molecules point one way or the other. Nonresonant second-order optical techniques could overcome these issues and be the method of choice for probing water orientation and flipping in response to applied potentials.

Consider the amphoteric nature of the oxides that terminate many electrodes used for the oxygen evolution reaction (OER). This reaction is typically carried out at high pH (*19*) where, at open circuit potential (OCP), many of the interfacial water molecules point their protons to the electrode surface. In this configuration, access of the electrode’s active sites to the electrons in water’s oxygen atoms would be blocked by water’s protons. An externally applied potential would need to be sufficiently high to weaken the interfacial hydrogen bond network so that the water molecules can flip to point their electron source (the oxygen atoms) toward the electrode’s active site (the high oxidation state metal oxo site). The energy associated with water flipping is a likely contributor to the water oxidation overpotential. Quantifying this work requires a quantification of the number of net-aligned Stern layer water molecules per unit area.

The sensitivity of nonlinear optical processes to interfacial structure and electrostatics should make it possible to quantify and track the number of Stern layer water molecules per unit area that are flipping, and the associated energetics, as a function of the applied potential, provided the strong absorber problem can be overcome [here, we work within the assumptions of the Stern layer as, for instance, described in Bard and Faulkner (*20*)]. Given the prominent role of water’s oxygen atoms as an earth-abundant electron source and the aforementioned need for water flipping to access them, quantifying how (i) the number of net-aligned water molecules, (ii) the electric field, and (iii) the Stern layer energy density depend on externally applied potential would add fundamental insights into our molecular understanding of electrochemical water oxidation. As we will show below, these three properties are readily accessible via the total interfacial potential, Φ_tot_, and the second-order nonlinear susceptibility, χ^(2)^, which we demonstrate here are both encoded in the experimental observables, namely the amplitude and phase of the second harmonic generation (SHG) response.

Besides resonant vibrational sum frequency generation studies of electrified interfaces [see, for instance, (*21*–*27*)] prior nonresonant nonlinear optical studies of electrode:electrolyte interfaces have largely been based on SHG intensity measurements [see Gruen’s (*28*) and Nagy and Roy’s (*29*) pioneering work on nickel electrodes]. These studies hark back to nonlinear electroreflectance studies from silver electrodes (please see note S1) (*30*–*32*). Recent approaches have focused on potential-of-zero charge quantifications via SHG amplitude and phase measurements on a platinum electrode (*9*). We now use optically transparent thin nickel nanolayers for which we quantify the Stern layer structure, the interfacial field, and the Stern layer energy density via $\Phi {\left(0\right)}_{\mathrm{tot}}$ and χ^(2)^.

## RESULTS

### Intensity measurements of electric field–induced SHG

**Fig. 1.**
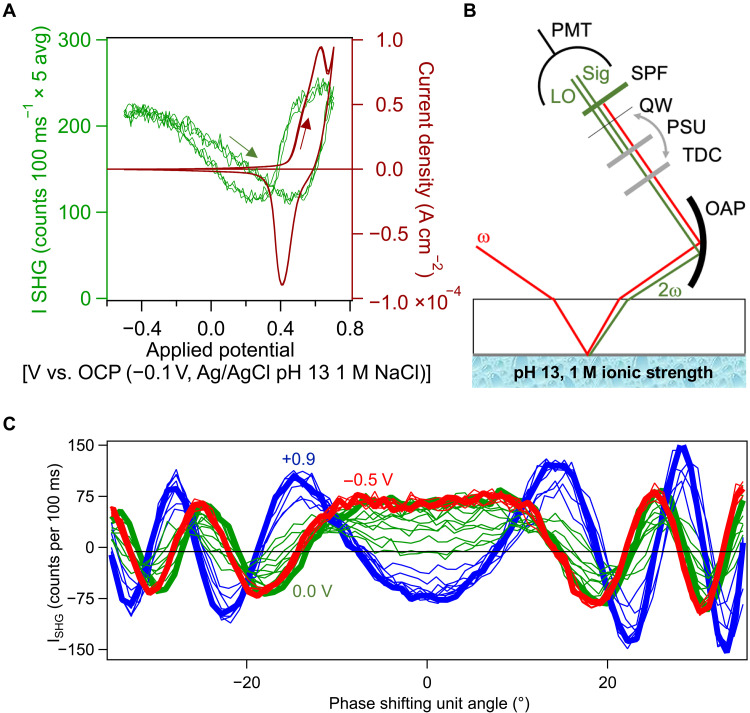
From intensity to amplitude and phase result SHG measurements at the electrolyte:electrode interface. (**A**) SHG intensity (left ordinate) and current density (right ordinate) recorded as a function of applied potential during three replicate cyclic voltammograms collected at 20 mV s^−1^. (**B**) Top view of the beam path for the SHG signal and local oscillator pair. OAP, off-axis parabolic mirror; TDC, time-delay compensator; PSU, motorized phase shifting unit; QW, quartz wafer; Sig, signal; LO, local oscillator; SPF, short-pass filter; PMT, photomultiplier tube. Beams offset for clarity. (**C**) Interference fringes recorded from the electrode:electrolyte interface as a function of applied potential, with 1 M NaClO_4_ and pH 13, adjusted using NaOH.

We begin with a 10-nm-thin nickel layer [5.1 ± 0.5–Å root mean square (RMS) roughness] prepared by physical vapor deposition on a glass microscope slide that is subsequently placed into a custom-designed spectro-electrochemical cell (please see fig. S1A and Materials and Methods) connected to an electrochemical workstation. Probing with a femtosecond laser oscillator (80 fs, 1034 nm, 75.5 MHz) and using single-photon counting, we find that the SHG intensity is quadratic in input power (fig. S1B). When recording the SHG intensity simultaneously with the current density as a function of applied potential at pH 13 (as well as pH 7, 9, and 11) and 1 M ionic strength (NaClO_4_ as well as alkali chlorides), we find SHG intensity minima that precede the peak potentials of the well-known Ni^2+^/Ni^3+^ redox pair (Fig. 1A), which follows the expected (scan rate)^1/2^ dependence (fig. S2, A and B) (*33*).

### SHG amplitude and phase measurements

To obtain the SHG amplitude and the absolute phase, we record SHG interference patterns generated by beating the SHG signals from two sources against one another (Fig. 1B): Source 1 is the electrode:electrolyte interface (producing the “signal”) and source 2 is a 50-μm-thin z-cut α-quartz wafer (producing the local oscillator, “LO”) (*34*). The sample and the α-quartz wafer bracket a phase shifting unit (PSU) consisting of a 1-mm-thin fused silica plate mounted on a computerized rotating stage. Collecting the SHG intensity as a function of the rotational angle of the PSU produces signal + LO interference fringes whose amplitudes and phases change as we vary the applied potential between −0.5 and +0.9 V versus Ag/AgCl (Fig. 1C). At each applied potential, the SHG amplitude and phase are obtained through a custom fit function (please see note S2).

### Estimate for the total interfacial potential and the second-order nonlinear susceptibility from the SHG amplitude and phase - normalization and referencing

**Fig. 2.**
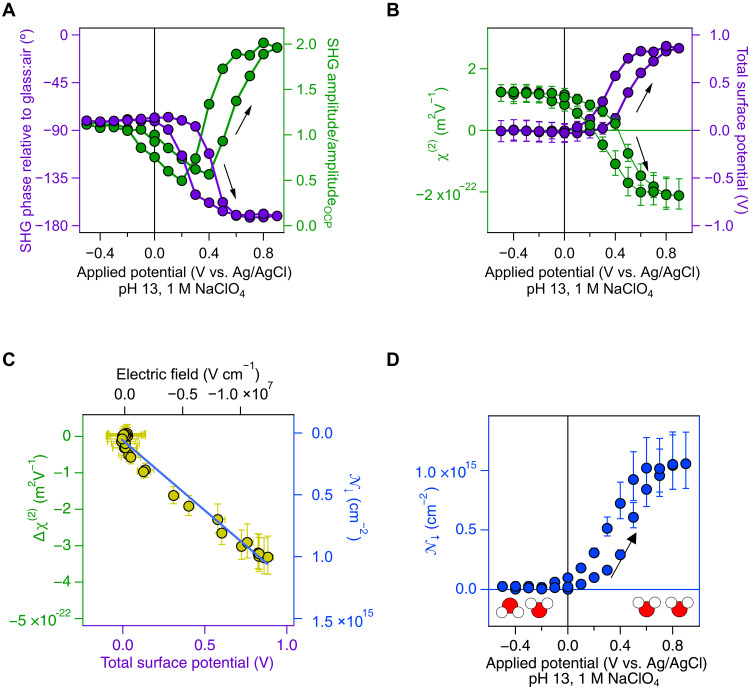
From second harmonic amplitude and phase to the total potential and second-order nonlinear susceptibility. (**A**) SHG phase (left ordinate) and amplitude (right ordinate) as a function of applied potential. Uncertainties in the fit parameters are 1.5° in the phase and 3% in the intensity and are obscured by the circle diameter. (**B**) Second-order nonlinear susceptibility (left ordinate) and total potential (right ordinate) as a function of applied potential. Uncertainties from propagating ±19° phase uncertainty. (**C**) Second-order nonlinear susceptibility at a given total surface potential minus the second-order nonlinear susceptibility obtained at OCP (left ordinate) and number of net-aligned Stern layer water molecules (right ordinate) as a function of total surface potential (lower abscissa) and electric field (upper abscissa). (**D**) Number of net-aligned Stern layer water molecules pointing their oxygen atoms toward the electrode as a function of applied potential.

Using the SHG amplitude and phase, we estimate the total interfacial potential, $\Phi {\left(0\right)}_{\mathrm{tot}}$, and the second-order nonlinear susceptibility, χ^(2)^. Using air as opposed to electrolyte, we first obtain the absolute zero phase from the uncoated portion of a glass microscope slide having one half coated with 10-nm nickel and then move the sample cell over by a few millimeters to determine the phase difference of the glass:air versus nickel:air interface to be = −76° ± 19° (SD obtained from Gaussian histogram analysis of 15 electrodes; please see the Supplementary Materials and fig. S3A). We then add electrolyte, determine the phase difference of the nickel:air versus nickel:electrolyte interface to be 4° ± 18°, and obtain the point estimates (and associated uncertainties) for the SHG signal phase, φ_sig_, at a given applied potential, Φ, from φ_sig_ = φ_fit,Φ_ − φ_fit,OCP_ − (76° ± 19°). We then normalize the SHG amplitude to the amplitude obtained at OCP. Figure 2A shows that φ_sig_ delays with increasing applied potential in a sigmoidal fashion, with a total phase change relative to OCP of −90° at +0.9 V applied. On the reverse scan, the phase advances back to 0°. The amplitude goes through minima at the applied potentials that coincide with the SHG intensity minima seen in Fig. 1A. The experiment reproduces reasonably well over seven different electrodes (fig. S3B). We calibrate the SHG response from our optical window against the second-order nonlinear susceptibility of another z-cut α-quartz piece put in place of the electrolyte solution (*34*, *35*), accounting for the normalization factor at OCP, the Fresnel coefficients, and the wave vector mismatch in our experimental geometry (please see figs. S4 and S5 and note S3). We then expand an optical model for quantifying $\Phi {\left(0\right)}_{\mathrm{tot}}$ and χ^(2)^ from the SHG amplitude and phase measured at silica:water interfaces for high ionic strength (*34*) to include the metal-specific contributions to the second-order nonlinear susceptibility discussed earlier by Guyot-Sionnest *et al.* (for Ag) (*36*) and Nagy and Roy (for Ni) (*29*). In other words, we now account for the metal phase shift and specific responses from the nickel surface in contact with the electrolyte. Furthermore, we use a bare glass window control to establish an absolute zero phase, given that $E_{\mathrm{SHG},\text{glass}:\mathrm{air}}e^{i\varphi_{,\text{glass}:\mathrm{air}}}=\text{real}$, with $\varphi_{,\text{glass}:\mathrm{air}}$ = 0° (please see the Supplementary Materials). We obtain the following expression for the total potential drop across the nickel electrode:electrolyte interface (see note S4)

$$\Phi {\left(0\right)}_{\mathrm{tot}}=-\frac{C\cdot E_{\mathrm{sig},\text{norm}}\left\{5\cdot \text{cos}\left(\varphi_{\mathrm{sig}}\right)+sin\left(\varphi_{\mathrm{sig}}\right)\right\}}{\left(5+1.5\right)\chi_{\text{water}}^{\left(3\right)}}$$ (1)

We note that Eq. 1 is specific to the nickel:electrolyte interface and is not transferable to other electrodes unless the required electrode phase shifts and specific electrode surface responses are determined or estimated. Here, *C* is the calibration factor that also accounts for OCP normalization of the measured SHG intensities and the Fresnel coefficients (*C* = 3.1 × 10^−22^ m^2^ V^−1^ in our case; please see note S3), *E_sig,norm_* is the measured SHG amplitude normalized to the value obtained at OCP (the condition at which we calibrate to quartz; see note S3), φ_sig_ is the phase relative to the zero phase from the glass:air interface, and $\chi_{\text{water}}^{\left(3\right)}$ is the third-order nonlinear susceptibility of the diffuse layer (1 × 10^−21^ m^2^ V^−2^ from experiment and theory) (*35*, *37*), which is invariant with ionic strength, pH, and surface composition (*38*). Equation 1 accounts for the ~five times larger nonlinear optical response we obtain from the nickel nanolayer when compared to a fused silica window, both at pH 13 and 1 M ionic strength. This experimentally determined factor of 5 is in excellent agreement with the computed factor of 4.5 in eq. 7 of Nagy and Roy and the one-half term in eq. 1 of Guyot-Sionnest *et al.* that account for metals’ bulk magnetic dipole contribution to $\chi^{\left(2\right)}$ (*29*, *36*). With Eq. 1 establishing $\Phi {\left(0\right)}_{\mathrm{tot}}$, the second-order nonlinear susceptibility is given by (see notes S4 and S5)

$$\chi^{\left(2\right)}=-\left\{C\cdot E_{\mathrm{sig},\text{norm}}\text{sin}\left(\varphi_{\mathrm{sig}}\right)+1.5\cdot \Phi {\left(0\right)}_{\mathrm{tot}}\cdot \chi_{\text{water}}^{\left(3\right)}\right\}/5$$ (2)

Again, Eq. 2 is specific to the nickel:electrolyte interface and not directly transferrable to other electrodes. An important aspect to consider here is that the up/down orientation of water is directly related to the sign of $\chi^{\left(2\right)}$ (*25*).

### Total interfacial potential and the second-order nonlinear susceptibility

Figure 2B shows the second-order nonlinear susceptibility and the total interfacial potential as a function of externally applied potential. From −0.4 V applied to 0 V applied, the total surface potential is near zero mV (± 100 mV from the −60° to −100° range in the phase relative to that of the glass:air interface), consistent with slight negative ζ-potentials at pH 11 and 12.5 of −12 and − 15 mV, respectively, in 0.1 M NaNO_3_ (points of zero charge of nickel oxides are at or below pH 11) (*39*–*41*). We note that the total potential is the sum of the Gouy-Chapman-Stern potential associated with the mobile charges (ions) and the contributions from the immobile charges (electrons bound to the molecules and ions), like from dipoles and quadrupoles (*42*). The total potential increases with increasingly positive applied potential until it plateaus near +0.8 V (±0.2 V uncertainty from the replicate electrode measurements; fig. S3C) at an applied potential of +0.9 V. We note that the absolute potential at an electrode:electrolyte interface cannot be measured using electrochemical means, which only provides the potential difference between two electrodes. The optical approach here does provide the total potential from a single electrode:electrolyte interface, similar to what is in principle possible with (notably slower) x-ray spectroscopic or electrical impedance measurements on field effect transistors (*43*–*48*).

Figure 2B also shows that at OCP, $\chi_{\mathrm{OCP}}^{\left(2\right)}$ is ~1.1 × 10^−22^ m^2^ V^−1^. This nonzero value is attributed to the net-aligned dipoles from the interfacial NiOH, NiO^−^, and NiOH_2_^+^ groups. Its positive value indicates a net “up” orientation of the interfacial dipoles, i.e., the positive end (Ni^2+^) pointing into the electrode and the negative end (OH, O^−^, or OH_2_^+^) pointing into the electrolyte. This interpretation is consistent with SHG results from colloidal (*37*) and macroscopically flat (*34*) surfaces showing that positively (resp., negatively) signed values of $\chi^{\left(2\right)}$ correspond to water dipoles pointing their negative end (oxygen) away from (resp., toward) the surface. Figure 2B indicates that the second-order nonlinear susceptibility becomes smaller in magnitude as the applied potential becomes more positive and that it crosses zero to become negatively signed at +0.4 V (resp., 0.2 V) applied potential on the forward (resp., reverse) scan, just where the SHG intensities goes through their minima (Fig. 1A). At +0.8 V applied, $\chi^{\left(2\right)}$ approaches −1.6 × 10^−22^ × 10^−22^ to −3.1 × 10^−22^ × 10^−22^ m^2^ V^−1^, about two times larger in magnitude, albeit oppositely signed, when compared to the value at OCP. Figure S3C shows that the χ^(2)^ and Φ(0)_tot_ estimates reproduce reasonably well over seven different electrodes, while fig. S3D shows no apparent pH dependence of the χ^(2)^ versus applied potential response between pH 13 and 7, while the Φ(0)_tot_ estimates increase to slightly more than 1 V at the highest positive potential applied, with a pH dependence of (−0.09 ± 0.04) V pH^−1^, encompassing the theoretical 0.059 V pH^−1^ Nernst slope at room temperature.

As the interfacial NiOH, NiO^−^, and NiOH_2_^+^ groups cannot flip their net orientation, we subtract $\chi_{\mathrm{OCP}}^{\left(2\right)}$ (1.1 × 10^−22^ × 10^−22^ m^2^ V^−1^) from the $\chi^{\left(2\right)}$ values obtained at each applied potential to compute the change in the second-order nonlinear susceptibility, $∆\chi^{\left(2\right)}$. The aim is to estimate the $\chi^{\left(2\right)}$ contribution from the mobile Stern lay water molecules, which can change their orientation distribution in response to the applied potential. When $\Phi {\left(0\right)}_{\mathrm{tot}}$ is zero, we find that $∆\chi^{\left(2\right)}$ is near zero (Fig. 2C), which indicates a largely isotropic arrangement of the Stern layer water molecules, in which an approximately equal number of interfacial water molecules point their dipole moments up versus down or are all fully disordered. In other words, 𝒩 · <α^(2)^> = 0, where 𝒩 is the total number of Stern layer water molecules and · <α^(2)^> is the water’s orientationally averaged molecular hyperpolarizability), consistent with the small negative ζ-potential at pH 12 (*39*).

### Number of net-aligned water molecules per square centimeter

We then proceeded to estimate the number of water molecules that flip their dipole orientations. To this end, we use the molecular hyperpolarizability for a liquid water model estimated by Gubskaya and Kusalik (*49*) at the MP2 and MP4 level of theory [α^(2)^ = 5.3 × 10^−52^ C m^3^ V^−2^]. This value was used recently by the Roke group (*37*) to estimate the nonresonant third-order nonlinear susceptibility, χ^(3)^, of liquid water, which is in good agreement with the experimental value reported by the Wen group (*35*). Dividing the $∆\chi^{\left(2\right)}$ values shown in Fig. 2C by α^(2)^ and multiplying by a Stern layer water permittivity estimate of 1.9 [the mean of ε = 1.77, the square of water’s index of refraction at 515 nm, and ε = 2.0, from recent experiments (*50*)] and the vacuum permittivity, ε_0_, according to 𝒩_↓_ = |$∆\chi^{\left(2\right)}$|·ε ε*_0_*/[10^4^ cm^2^ m^−2^·α^(2)^] yields the number of water molecules per square centimeter that point their oxygen atoms down toward the electrode. Figure 2D shows that at the most positive applied potential (+0.9 V), ~1.1 × 10^15^ water molecules/cm^2^ have a net orientation with their oxygen atoms pointing toward the electrode. Larger values for 𝒩_↓_ would arise from larger values for the Stern layer relative permittivity. In addition, the angular orientation distribution of the Stern layer water molecules relative to the surface normal is not known and could very well be multimodal. Therefore, our estimate for the number of net-aligned water molecules in the Stern layer represents an upper limit. Under the s-in/p-out polarization combination used here, our estimate for 𝒩_↓, max_ = 1.1 × 10^15^ cm^−2^ matches the geometric number density of water molecules on the surface of a 1-cm^3^ cube of liquid water at standard temperature and pressure (1 × 10^15^ cm^−2^), i.e., one water monolayer, consistent with the notion that the experiments report, to leading order, on the surface normal projection of the dipole orientations. We also note that recent atomistic simulations of water at electrified graphene interfaces show a somewhat higher Stern layer density (~1.8 to 2.7 g cm^−3^) as the applied potential goes from 0 to 2.5 V (*1*), corresponding to 1.5 to 2.7 × 10^15^ net-aligned water molecules cm^−2^ (or up to about two monolayers) when using the experimentally derived $∆\chi^{\left(2\right)}$ point estimates reported here. We also note in note S6 and the figures therein that vibrational sum frequency generation spectra recorded under electrochemical control show potential-dependent responses but do not provide the number of net-aligned Stern layer water molecules due to complications discussed in that section. They do, however, provide evidence for a tight linear correlation between the nonresonant SHG amplitude reported here and the vibrational sum frequency generation response at each applied potential. Moreover, the vibrational sum frequency generation response at each applied potential scales linearly with the total potential derived from the phase-resolved SHG measurements reported here and correlates closely (to within 20%) with the number of net-aligned Stern layer water molecules.

### 2D two-state Ising model

**Fig. 3.**
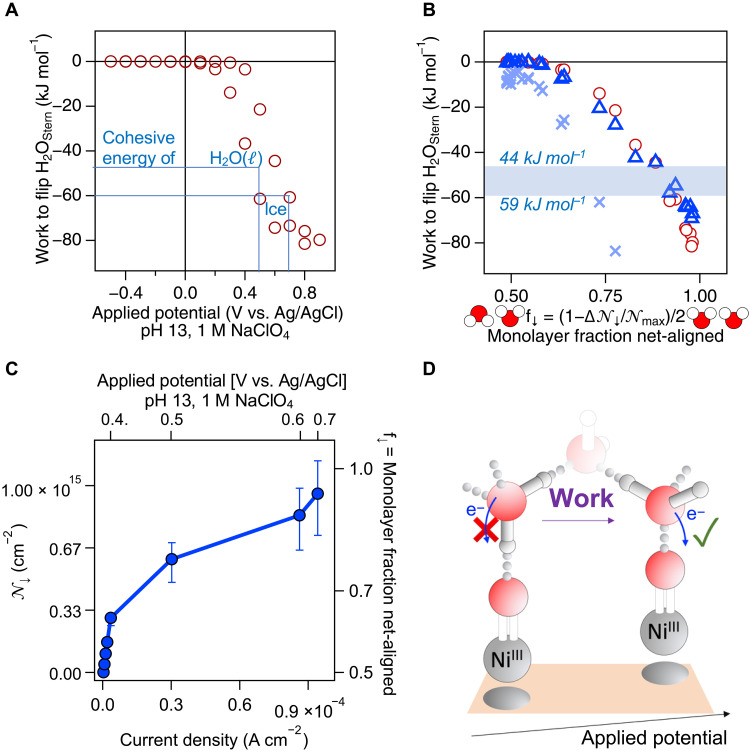
Connecting the work associated with water flipping with electrochemical performance. (**A**) Work associated with Stern layer water flipping as a function of applied potential. The cohesive energies of liquid water and ice are indicated. (**B**) Work associated with Stern layer water flipping as a function of the fraction of water molecules having a net-orientation with their oxygen atoms pointed toward the electrode. Upper and lower bounds of shaded area indicate range of cohesive energies of liquid water and ice, respectively. Dark blue triangles and light blue x’s indicate 2D-Ising model result with *J* = −1 × 10^−20^ J and *J* = −1 × 10^−19^ J, respectively. (**C**) Number (left ordinate) and fraction (right ordinate) of net-aligned water molecules pointing their oxygen atoms toward the electrode as a function of measured current density (bottom abscissa) and applied potential (top abscissa). (**D**) Cartoon of water flipping concept.

In contrast to the sigmoidal dependence of the number of net-aligned Stern layer water molecules on applied potential shown in Fig. 2D, the right-hand axis of Fig. 2C shows that water flipping is linear in the total potential across the electrode:electrolyte interface. Flipping all the Stern layer water molecules requires a field of close to −1 × 10^7^ V cm^−1^ (top *x* axis in Fig. 2C)—an experimental match with estimates from classic electrochemical textbooks (*20*), now obtained using purely optical means. To investigate the energetics associated with Stern layer water flipping, we computed the total energy density in the Stern layer by multiplying the total potential by the elemental charge and the number of oriented Stern layer water molecules according to *E_flip_* = Φ_tot_^.^e^.^𝒩_water↓_, showing a sigmoidal variation with applied potential (Fig. 3A). In contrast, we find a parabolic variation of the Stern layer energy density with *f*_↓_, the fraction of Stern layer water molecules pointing their oxygen atoms toward the electrode (Fig. 3B; note that for *f*_↓_ = 0.5, 𝒩_↓_ = 0, i.e., there is no net order, and for *f*_↓_ = 1.0, 𝒩_↓_ = 𝒩_↓,max_, i.e., all water molecules have flipped). The experimental results can be interpreted using a two-dimensional (2D), two-state Ising model in which we express *F*, the Helmholtz free energy mean field solution per water molecule for the square lattice model (*z* = 4), according to (*51*)

$$F={\left({𝒩}_{↓}\right)}^{2}J\frac{z}{2}-\beta^{-1}\text{ln}\left\{\text{cosh}\left[\beta \cdot \left(Jz|{𝒩}_{↓}|+e\cdot \Phi_{tot}\right)\right]\right\}$$ (3)

Here, ${𝒩}_{↓}=-3\times 10^{-2}-\Phi_{\mathrm{tot}}\times 1\;V^{-1}$ (the linear least squares fit result of 𝒩_↓_ versus Φ_*tot*_, Fig. 2C, scaled by 10^−15^ to a per-molecule basis), β = (*k*_*B*_*T*)^−1^ with *k*_*B*_ being the Boltzmann constant, *T* is the temperature (300 K), and *e* is the elementary charge. The model recapitulates the experimental data with a coupling constant, *J*, of −1 × 10^−20^ J per water molecule (6 kJ mol^−1^), again using ε = 1.9. Increasing *J* tenfold increases the magnitude of the work associated with water flipping considerably. These results support the notion that the experiments are largely sensitive to the dipole up versus “down” orientations of the Stern layer water molecules. At the highest applied potential, where 𝒩_↓_ = 𝒩__↓, max__ = 1 × 10^15^ molecules, the work associated with water flipping corresponds to 80 kJ mol^−1^, exceeding the cohesive energy of ice by 20 kJ mol^−1^ (*52*). Figure 3C shows that the water flipping process begins before the onset of Faradaic current flow (the nickel oxidation wave at +0.4 V), indicating that the OER in this case requires water flipping first, followed by electron transfer.

## DISCUSSION

The facile nonlinear optical readout during electrochemical processes reported here allows us to estimate the number of Stern layer water molecules that point their electron-rich oxygen atoms toward an anode as a function of applied potential. Our estimates for the total surface potential, the electric field, and the work in the Stern layer under operando electrochemical conditions indicate that at the highest potentials applied (+0.9 V versus Ag/AgCl, pH 13), about 1.1 × 10^15^ water molecules/cm^2^ are net-aligned oxygen atoms toward the electrode. A 2D two-state Ising model recapitulates the experimental results. Before starting the Faradaic process at +0.4 V, the work associated with water flipping is negligible, but approximately two-thirds to three-fourths of a monolayer of Stern layer water molecules are already net-aligned with their oxygen atoms pointed toward the electrode. These results indicate that water orientation is a necessary condition for the OER to occur in the case of the nickel electrodes studied here (Fig. 3D). At +0.6 V applied potential, the Stern layer energy density has increased to match the cohesive energy of liquid water. At ~ +0.9 V applied potential, all the Stern layer water molecules (𝒩_↓, max_ = 1.1 × 10^15^ cm^−2^) have flipped to point their oxygen atoms toward the electrode, and the associated Stern layer energy exceeds the 59 kJ mol^−1^ cohesive energy of ice by ~20 kJ mol^−1^.

We caution that the optical model presented here is specific for nickel electrodes and not directly transferrable to other electrodes. We expect that our fundamental insights will add to the ongoing rapid development of molecular electrochemistry. This work presents the idea that the orientation of interfacial water molecules is important because it is in water’s oxygen atoms where the electrons desired in the OER are located. If access of the electrode’s active sites to water’s oxygen is blocked by water’s hydrogen atoms (“water’s oxygen pointed away from the surface”), then the potential needs to be driven up for water flipping to occur. Noting that the level of quantification presented here would not be possible with the information and models that have existed until now, the experimental data we present can serve as benchmarks for theoretical models of the electrical double layer and electrochemistry. They establish that the Stern layer water molecules flip before the onset of the OER and thereby open the possibility to pursue the energy barrier for water flipping as a means for addressing the OER’s high overpotential on nickel anodes. Beyond the physical insights and experimental benchmarks, the ability to (i) count the number of Stern layer water molecules, (ii) determine their net absolute orientation, and (iii) quantify the electrostatic field and energy density at electrode:electrolyte interfaces under operando conditions represents a diagnostic toolkit that we envision to help elucidate why the platinum group elements are better water oxidation catalysts when compared to, say, catalysts composed of earth abundant metals such as nickel or iron, particularly from a perspective of the electron source, i.e., that of the interfacial water molecules. Our capabilities and insights into solvent structure and energetics should be equally applicable to the ongoing development of synthetic organic electrochemistry (*53*).

## MATERIALS AND METHODS

The electrochemical workstation is a Metrohm Autolab model (PGSTAT302N with a SCAN250 true linear analog sweep module). The nickel (working), counter (platinum), and reference (Ag:AgCl) electrodes in contact with aqueous electrolyte (pH 13 and 1 M NaClO_4_). FKM O-rings are used for sealing the spectro-electrochemical cell housing the electrodes, which is unstirred and consists of a double-paned custom-designed assembly (redox.me) manufactured from PEEK (please see fig. S1A). The OCP is measured before each electrochemical experiment to be −0.094 ± 0.007 V (versus Ag/AgCl, at pH 13, 1 M ionic strength, average of 33 measurements). One window consists of a standard 1 in–by–3 in. (2.54 cm–by–7.62 cm) VWR microscope glass slide onto which a 10-nm-thin nickel nanolayer is deposited from nickel sources having a purity of 99.98 (Kurt J. Lesker) using a physical vapor deposition method that minimizes the presence of low-boiling point impurities (K, Ca, and Mg) in the deposited nanolayer (*54*). The second window is a fused silica window that allows for the incident laser pulses at the fundamental frequency to exit the electrochemical assembly toward a beam stop.

X-ray photoelectron spectroscopy shows the presence of nickel oxide on the electrode surface (*54*). The nickel oxidation and reduction waves integrate to between 1.1 × 10^−3^ and 1.3 × 10^−3^ C, corresponding to 7 × 10^15^ to 8 × 10^15^ electrons transferred (comparable to undoped NiO electrodes) (*55*) and thus 7 to 8 monolayer equivalents of Ni^2+^/Ni^3+^, or an electrochemically active oxide thickness of 1.4 to 1.6 nm, assuming a Ni─O bond length of 2 Å. This oxide thickness is on the order of what we reported earlier from atom probe tomography for iron nanolayers (*56*) prepared using the same low-impurity physical vapor deposition (PVD) method that is used here for the nickel nanolayers. Atomic force microscopy (Bruker) shows the electrodes to have an RMS roughness of 5.1 ± 0.5 Å over 1 μm–by–1 μm and 300 nm–by–300 nm areas before and after the electrochemical measurements (please see fig. S6), matching that of the substrates onto which they are deposited. Optical imaging shows no pinholes, but they are found on rare occasions in scanning electron microscopy images (please see fig. S7). Water contact angles recorded immediately after nanolayer formation are <10° but increase to more than 50° when the nanolayers are left in ambient laboratory air for several hundred hours (please see fig. S8), indicating hydrocarbon buildup. We therefore subject each electrode, once mounted in the e-chem cell, to replicate cyclic voltammograms (−0.3 to +0.8 V) until they are indistinguishable from one another, typically 5 to 10 cycles.

We direct 0.2 W from a LightConversion Flint oscillator (model FL1-02) producing 80-fs pulses at 1034 nm at a 75.5-MHz repetition rate onto the electrode:electrolyte interface using a defocused (−1 cm) 10-cm lens (spot size, ~100-μm diameter) and block the reflected fundamental light from the external air:window interface. We direct the SHG signal pulses along with the reflected fundamental pulses from the electrode:electrolyte interface toward an off-axis parabolic mirror and a 0.5-mm-thin uncoated calcite time delay compensator (Newlight Photonics, CAL12050-A) to account for spatial and temporal dispersion at the detector, as described in our earlier work (*34*). The fundamental and SHG pulses are then sent through a 1-mm-thin fused silica PSU(Edmund Optics) on a rotating stage (Standa model 8MR174-11) and then through a 50-μm-thin z-cut a-quartz wafer (Precision Micro-Optics PWQB-368252) producing the LO. The SHG pulse pair (signal + LO) then interferes at the detector (Hamamatsu, H8259-01) as a function of the PSU angle.

At each PSU angle, we collect the SHG signal at 100-ms acquisition time for 5, 10, or 20 s, so it takes as little as a minute to record one fringe and reset the PSU motor position. We use an applied voltage staircase in 100-mV steps that parks at a given voltage for the time required to record three fringes, of which we use the third for fitting. We then obtain the signal (i.e., electrode:electrolyte interface) amplitude and phase from a trigonometric fit function detailed in note S2.

Replicate measurements of the SHG phase in air were performed using various laser spot positions on a given glass slide or nickel electrode with 15 in different electrodes so as to account for variations in the measurements that come along with slight variations in how the sample cell is assembled and mounted between replicates/trials (please see fig. S3A). We first obtain the absolute zero phase from the uncoated portion of a glass microscope slide having one half coated with 10-nm nickel and then move the sample cell over by a few millimeters to determine the fitted phase of the metal nanolayer. We mount the slide in the electrochemical cell and contact it with air (no electrolyte present). The uncoated part of the glass slide is fully transparent at the wavelengths used here, not birefringent, and the surface potential is zero so that the SHG response is purely real, i.e., $E_{\mathrm{SHG},\text{glass}:\mathrm{air}}e^{i\varphi_{,\text{glass}:\mathrm{air}}}=\text{real}$, with $\varphi_{,\text{glass}:\mathrm{air}}=0{}^{\circ}$. We collect a fringe on the uncoated glass side and obtain a fitted phase, φ*_fit_*, using a trigonometric fit function detailed in note S2. This fitted phase is the offset we apply to the phases we obtain when moving the cell such that the laser beam hits the nickel nanolayer. Replicate measurements obtained by assembling and reassembling 15 glass/nickel slides in our cell show φ*_nickel:air_* − φ*_glass:air_* = −76° ± 19° (SD obtained from Gaussian histogram analysis; please see fig. S3A), with the upper limit close to the 90° phase shift reported for nonresonant sum frequency signals from gold (*57*). Keeping the laser focused on the nickel portion of the cell, we fill the cell with electrolyte using a peristaltic pump and obtain φ*_nickel:air_* − φ*_nickel:electrolyte_* = 4° ± 18° (again with 15 replicates) at OCP (−0.1 V versus Ag/AgCl in our cell; please see fig. S4). Given these results, we first offset the fitted phase obtained at each applied potential by the one obtained at OCP. We then subtract another 57° to 95° to estimate the absolute phase from the electrode:electrolyte interface under applied potential.

Attempts to obtain the absolute phase from a z-cut α-quartz crystal aligned along the *x* axis (*58*, *59*) and pressed against a glass slide in our electrochemical cell were unsuccessful. While the interference fringes are readily observed, the fitted phases vary tens to 100 s of degrees as we move from one sample spot to the next or from one sample assembly to the next. The problem persists with index-matching fluid. We attribute this result to imperfect flatness and the resulting gap between the two solids and conclude that the air-first and electrolyte-second approach is a reliable means for the phase estimate.

## Supplementary Materials

### This PDF file includes:

Figs. S1 to S10 Supplementary Notes S1 to S6 References
