# Supplementary material for: Quantifying Stern layer water alignment before and during the oxygen evolution reaction
Source: Sci Adv. 2025 Mar 5;11(10):eado8536. doi: 10.1126/sciadv.ado8536 (PMC11881897; doi:10.1126/sciadv.ado8536)
Supplement: Supplementary file 1 — Figs. S1 to S10 Supplementary Notes S1 to S6 References [file sciadv.ado8536_sm.pdf]

Science Advances
AAAS

# Supplementary Materials for

## Quantifying Stern layer water alignment before and during the oxygen evolution reaction

Raiden Speelman *et al.*

Corresponding author: Franz M. Geiger, f-geiger@northwestern.edu

*Sci. Adv.* **11**, eado8536 (2025)
DOI: 10.1126/sciadv.ado8536

**This PDF file includes:**

Figs. S1 to S10
Supplementary Notes S1 to S6
References

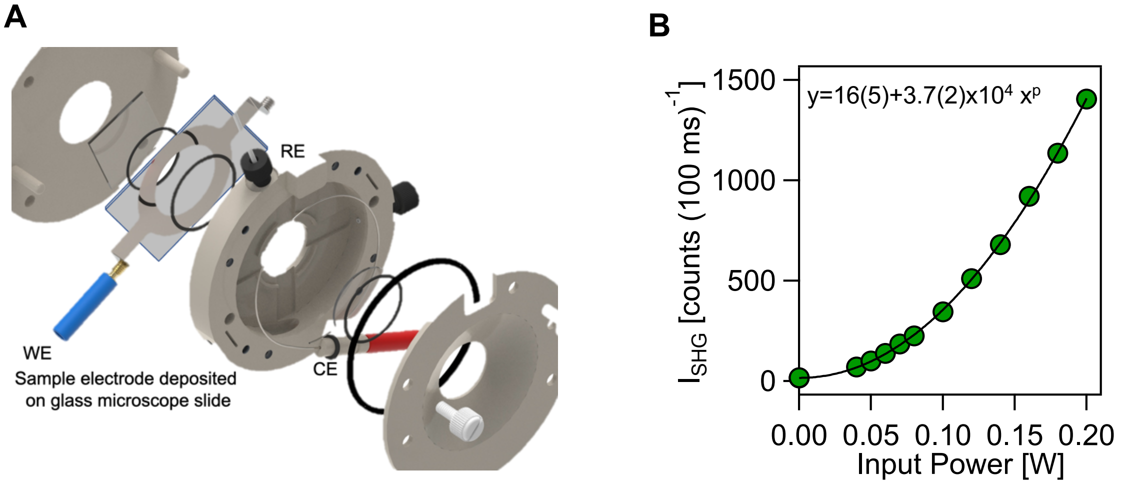


**Fig. S1. Experimental design. A**) Detailed view of the electrochemical cell used in the experiments. WE=working electrode, CE=platinum counter electrode, RE=Ag/AgCl reference electrode. **B**) SHG Signal intensity vs incident input power and fit to a power function producing an estimate for p of 2.04 ± 0.03.

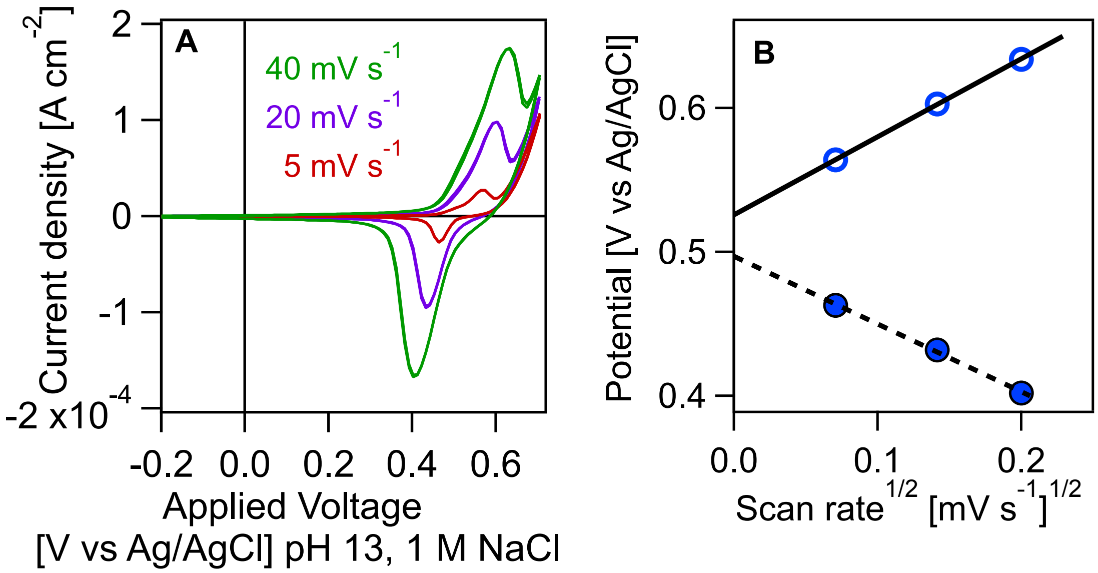


**Fig. S2. Electrochemical measurements. A**) Cyclic voltammograms recorded with indicated scan rates. **B**) Ni(II)/Ni(III) Oxidation peak potential vs (scan rate)$^{1/2}$ and linear fits (y=a+b*x)

where a=0.5259 ± 0.0009 V and 0.497 ± 0.003 V and b=0.542 ± 0.006 $mV^{1/2}s^{1/2}$ and -0.47 ± 0.02 $mV^{1/2}s^{1/2}$ for the anodic and the cathodic peaks, respectively.

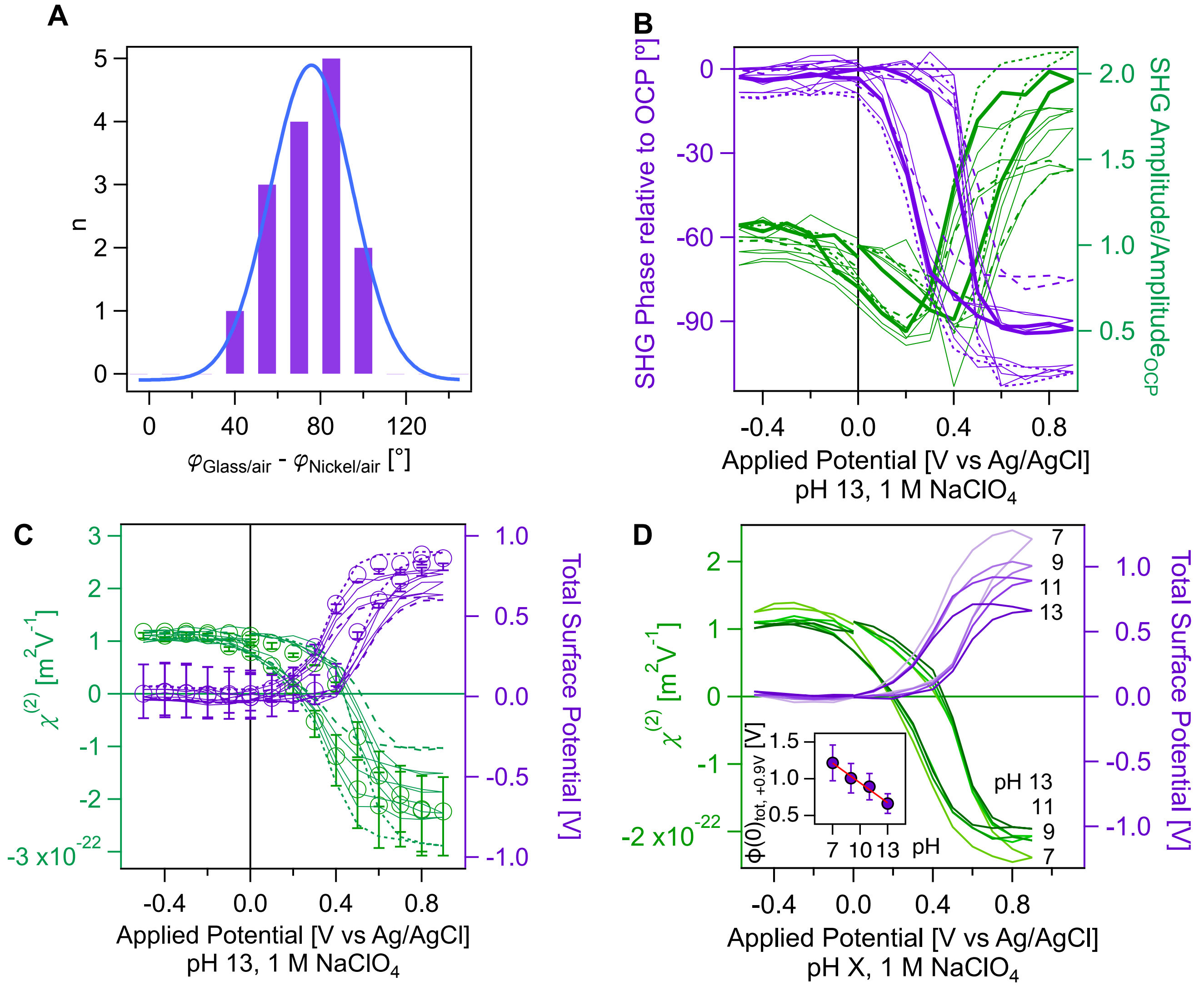


**Fig. S3. Experimental reproducibility. A**) Histogram of 15 measurements of $\varphi_{glass:air}$ - $\varphi_{nickel:air}$ and Gaussian fit having a mean of 76° and a standard deviation of 19°. **B**) SHG Amplitude and phase obtained from 7 different electrodes. As 3 examples, the thick, dash ed, and dotted lines m ark the amplitude and phase pairs obtained from a given electrode. **C**) Same as in b), but for $\chi^{(2)}$ and $\Phi(0)_{tot}$. The $\chi^{(2)}$ $\Phi(0)_{tot}$ pair from the main text is given as well (empty circles). **D**) Second-order nonlinear susceptibility and total interfacial potential as a function of applied potential for

electrolyte held at pH 7, 9, 11, and 13, indicated by increasingly darker line color. All pH runs performed on the same electrode; pH was changed using a peristaltic pump. Inset: $\Phi(0)_{tot}$ at +0.9V applied vs pH, and slope of 0.09 ±0.04 V $pH^{-1}$ from weighted linear least squares fit (solid line).

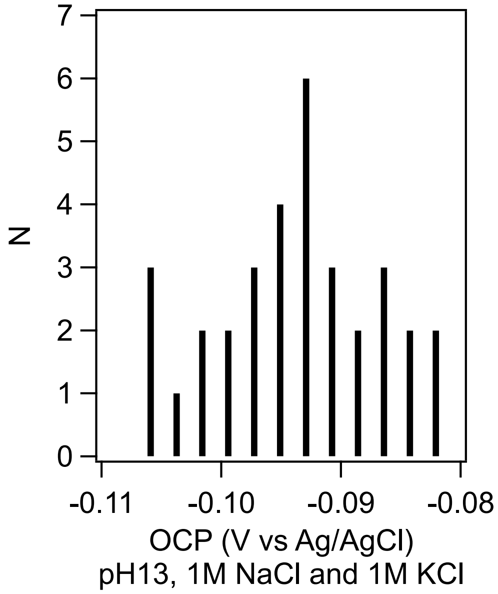


**Fig. S4. Histogram of open circuit potential before and after CV sweeps.** Data from 33 replicates.

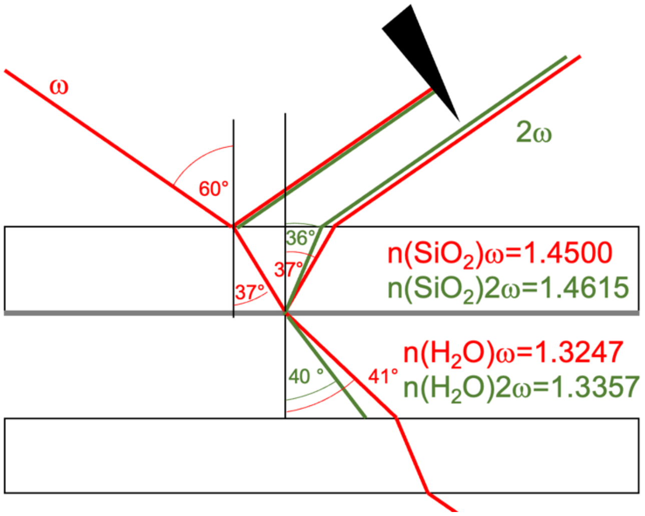

**Fig. S5. Optical layout.** Top view of the optical beam paths in the electrochemical cell. The 10 nm-thin nickel electrode is indicated as the thin grey line at the bottom of the top window. A piece of anodized aluminum blocks the reflected fundamental and second harmonic from the top surface of the top window.

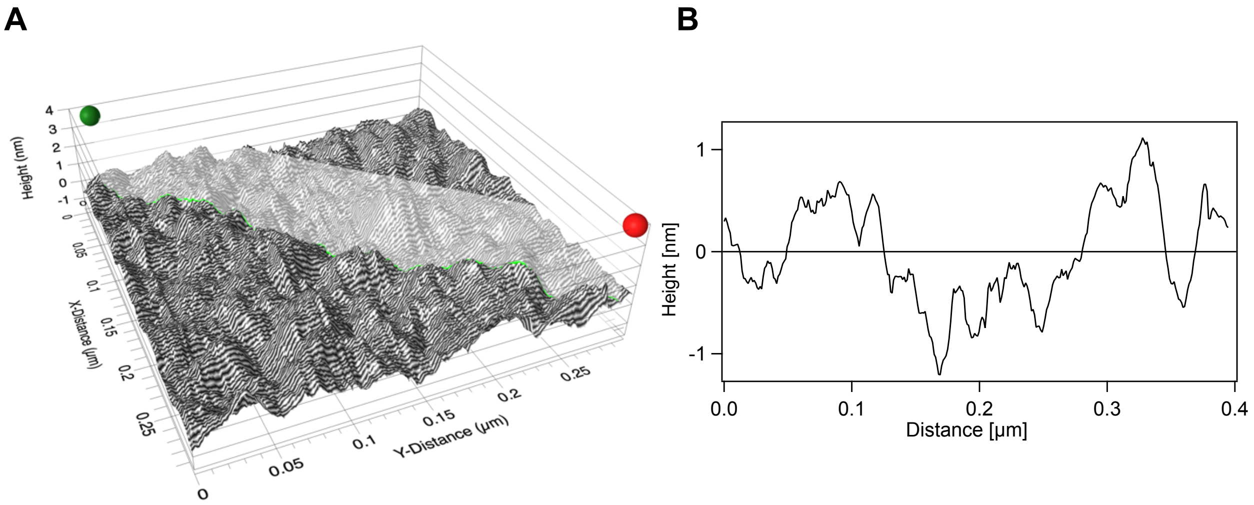


**Fig. S6. Atomic force microscopy. A**) Atomic force microscope image (Bruker Icon, tapping-in-air mode, 2 Hz scan rate) of a 10 nm nickel electrode on a VWR glass microscope slide after cyclic voltammetry. **B**) Line profile along the diagonal indicated in **A**).

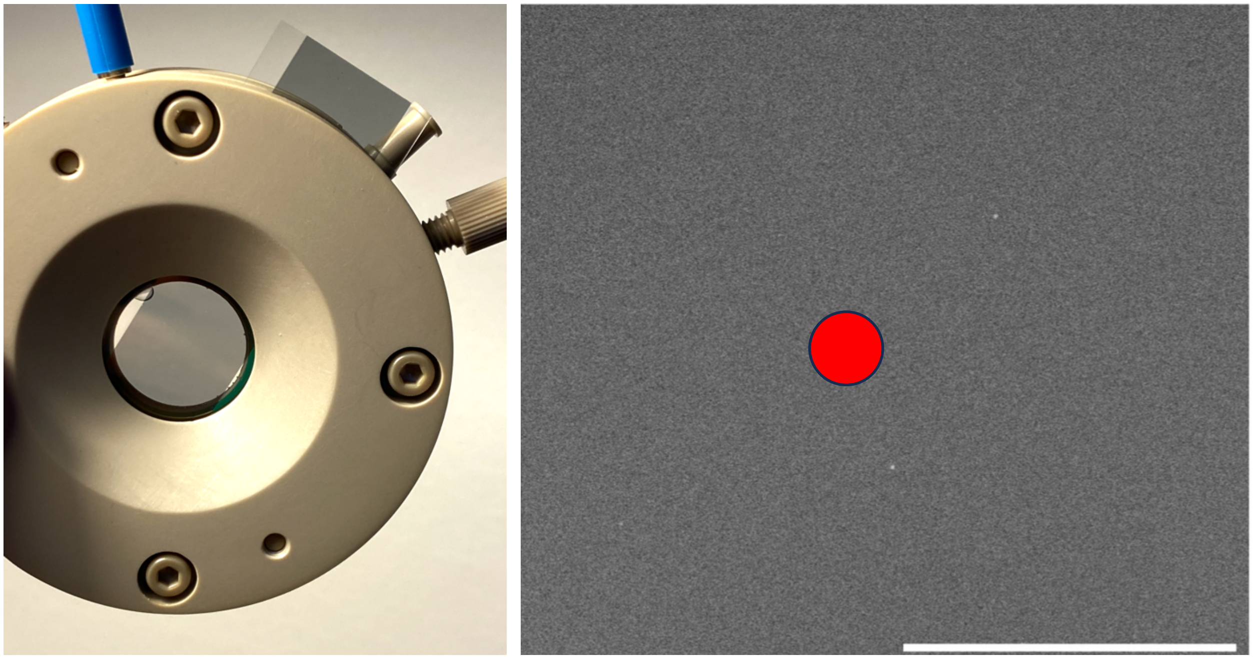

**Fig. S7. Optical image and scanning electron microscopy. left**) Optical image of a mounted 10 nm thin nickel nanolayer on a standard 3 x 1 $in^2$ VWR glass microscope slide. **right**) Scanning electron microscope image of a thin nickel nanolayer showing what appears to be three small pinholes that are observed on rare occasion. The red circle indicates the approximate laser spot size (100 µm). Scale bar=500 µm.

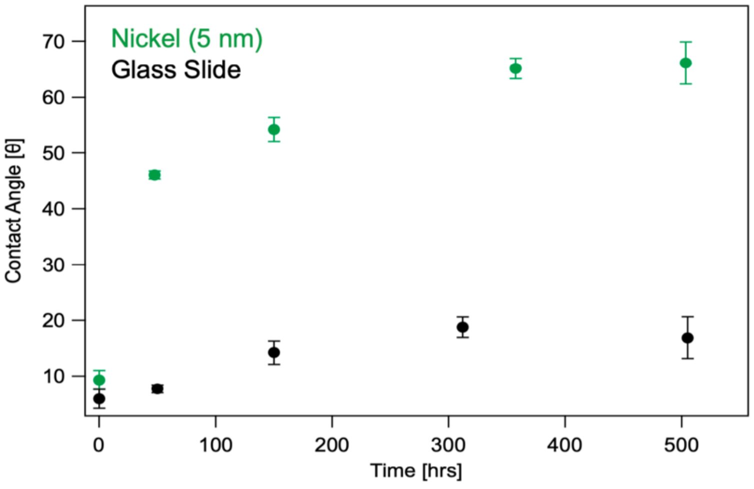


**Fig. S8. Contact angle measurements.** Water contact angles measured on nickel nanolayers (green) and uncoated glass slides (black) left in ambient laboratory air for the number of hours indicated.

**Supplementary Note S1.** SHG signals from aqueous electric double layers were first reported by Wang in 1969 (*60*). Heinz and Shen employed electrochemical conditions (*61, 62*), Richmond (*31, 63-67*), Corn (*32, 68*), and Guyot-Sionnest (*69*) pioneered the method in chemistry as electric-field induced second harmonic, and Eisenthal established it for insulators (*70*). The field grew(*71-74*) to include vibrational sum frequency generation (SFG) spectroscopy (*24, 26, 75-79*). Homodyne-detected SFG spectroscopy and SHG microscopy imaging under electrochemical control have now been realized by the Campen and Roke groups for Au electrodes in the electrochemical stability window as well as for the OER (*23, 80-82*). Liu and Shen reported phase-resolved nonlinear optical measurements of optically thin, gate-controlled Si:SiOx:water interfaces (*83*), while the Suntivich group applied phase-sensitive SHG to Pt electrodes to identify potentials of zero charge (*9, 84*).

**Supplementary Note S2.** We employ a rotating phase shifting unit based on the design by Huang

and Lewis (*85*). The interference fringes are fit to the following function, taken from Stolle et al. (*86*):

$$f(\theta) = a + b \cdot (\theta+\delta\theta) + c \cdot (\theta+\delta\theta)^2 + Esig \cdot \cos\{4\pi \cdot [0.00107566/(1.03 \cdot 10^{-6})] \cdot \{1.4619 \cdot \cos[\mathrm{asin}(\sin((\theta+\delta\theta)/1.4619)] - 1.4501 \cdot \cos[\mathrm{asin}(\sin((\theta+\delta\theta)/1.4501)]\} + \varphi_{fit}\} \quad \text{eqn. S1}$$

Here, a is y-axis offset, b and c account for the parabolic profile of the fringes that is due to slight reflection losses at increased angles, θ, of the phase shifting unit (PSU angle, varied by ±40° around 0°), δθ accounts for not being able to mount the PSU to be exactly perpendicular to the entering beams (the true 0°), $E_{sig}$ is the SHG amplitude, the factor 0.00107566 is the value of the fused silica plate thickness from replicate caliper measurements (in meters), $1.03 \cdot 10^{-6}$ is the value of the fundamental wavelength (in meters), 1.4619 and 1.4501 are the refractive indices of fused silica at the second harmonic and fundamental wavelength obtained from a four-parameter Cauchy equation fit to tabulated IR grade fused silica values available from ISP Optics, and $\varphi_{fit}$ is the fitted SHG phase. Eqn. S1 is fit to each fringe, with a, b, c, δθ, $E_{sig}$ and $\varphi_{fit}$ as fit parameters. Uncertainties from the IgorPro fitting algorithm are <3% in $E_{sig}$ and <1.5° in $\varphi_{sig}$. This technique has also been successfully applied to determine the nonlinear optical phase of gold surfaces in vibrational sum frequency spectroscopy (*86*).

**Supplementary Note S3.** We obtain the calibration factor, *C*, used in eqns. 1 and 2 as follows: We first measured the SHG intensity from a nickel electrode at pH 13 and 1 M NaCl at OCP (determined to be -0.1 V vs Ag/AgCl using the Autolab OCP-determination program sequence) to be 200 counts per 100 ms, with 0.2 mW input power and the -1 cm defocused lens arrangement described in the main text (see Fig. 1B in the main text). We then replaced the aqueous solution with a piece of z-cut α-quartz aligned as described previously (*34, 87*) and affixed to a glass

microscope slide (no Ni present) using a drop of index matching fluid. The resulting SHG intensity saturated the detector, so we reduced the input energy of the fundamental to 0.1 W. The resulting SHG intensity was measured to be 400,000 counts per 100 ms. Dividing the SHG intensity from the nickel:electrolyte interface measured before by a factor of 4 and taking square roots to obtain the electric field response difference resulted in a factor $E_{sample}/E_{quartz}$=1/90=0.011.

We then compute the effective second-order nonlinear susceptibility of the α-quartz piece sampled in our optical setup by dividing its bulk second-order nonlinear susceptibility by the wavevector mismatch (2.2 x $10^7$ $m^{-1}$ in our setup, using the angles and optical constants indicated in Fig. S5) to obtain a $\chi^{(2)}_{eff,quartz}$=3.0 x $10^{-22}$ $m^2V^{-1}$. Multiplying this value by the ratio of the Fresnel coefficients in our window/quartz vs window/electrolyte interface (computed as previously described (*34, 87*) using the angles indicated in Fig. S5, this ratio is 0.58/0.68) yields an estimate for *C* of 3.1 x $10^{-22}$ $m^2V^{-1}$. The calibration factor *C* (and therefore the Fresnel coefficients) can vary by up to +/- 20 percent and still produce $\chi^{(2)}$ point estimates (and thus Stern layer water numbers) that fall within the error bars we provide in Fig. 2. The inputs into the Fresnel coefficients are fundamental constants and measured properties like the refractive indices, with the one experimentally unknown value being the interfacial refractive index that is needed to compute $L_{zz}$, which can be approximated by as described by Shen and coworkers (*88*) and Liljeblad and Tyrode (*89*)). A +/- 20 percent uncertainty on that value is probably reasonable.

We note that the model described above treats the ten-nanometer thin nickel:nickel oxide as non-refractory, which experiments confirm that verify the lack of spatial displacement of a visible laser beam passing through the glass slide/nickel electrode as opposed to an uncoated portion of the electrode. We also treat the optical absorbance of the nickel nanolayer at the fundamental and the second harmonic (<20%) to be minor.

**Supplementary Note S4.** We express the nonlinear optical response from the electrode:electrolyte interface as follows:

$$C \cdot E_{sig,norm} e^{i\varphi_{sig}} = \chi^{(2)} - 5i\chi^{(2)} - \chi_w^{(3)} \Phi(0)_{tot}(1 + 1.5i) \qquad \text{eqn. S2}$$

Here, $C$ is the calibration factor estimated as described above, $E_{sig,norm}$ is the measured SHG amplitude normalized to the value obtained at zero applied volt (OCP, the condition at which we calibrate to quartz, as described above), and $\varphi_{sig} = \varphi_{fit,\Phi} - \varphi_{fit,OCP}$ - 76° ± 19°, as described in the main text. On the right-hand-side of the equation we have $\chi^{(2)}$, the second-order nonlinear susceptibility, the ca. 5-fold larger resonant contribution and its phase of -90° ($e^{-i\pi} = -1$, within the range of the measured 76° ± 19° glass:air to nickel:air phase difference described in the main text), the third-order nonlinear susceptibility of water, $\chi_w^{(3)}$, and the total interfacial potential, $\Phi(0)_{tot}$. We express the left-hand-side of eqn. S1 using the Euler identity and collect the real and imaginary terms to obtain

$$C \cdot E_{sig,norm} cos(\varphi_{sig}) = \chi^{(2)} - \chi_w^{(3)} \Phi(0)_{tot} \qquad \text{eqn. S3}$$

$$C \cdot E_{sig,norm} sin(\varphi_{sig}) = -5\chi^{(2)} - 1.5\chi_w^{(3)} \Phi(0)_{tot} \qquad \text{eqn. S4}$$

Eqn. 3 is rearranged for $\chi^{(2)}$ to yield

$$\chi^{(2)} = -\frac{C \cdot E_{sig,norm} sin(\varphi_{sig}) + 1.5\chi_w^{(3)} \Phi(0)_{tot}}{5} \qquad \text{eqn. S5}$$

Placing this expression for $\chi^{(2)}$ into eqn. S2 then yields the total interfacial potential as

$$\Phi(0)_{tot} = -\frac{C \cdot E_{sig,norm} \{5 \cdot cos(\varphi_{sig}) + sin(\varphi_{sig})\}}{(5+1.5)\chi_{water}^{(3)}} \qquad \text{eqn. S6}$$

**Supplementary Note S5.** The second-order nonlinear susceptibility and (eqn. S4) and especially the total potential (eqn. S5) vary little if the factor 5 in eqn. S4 is changed by ±1 to 4 or 6 (Fig. S9).

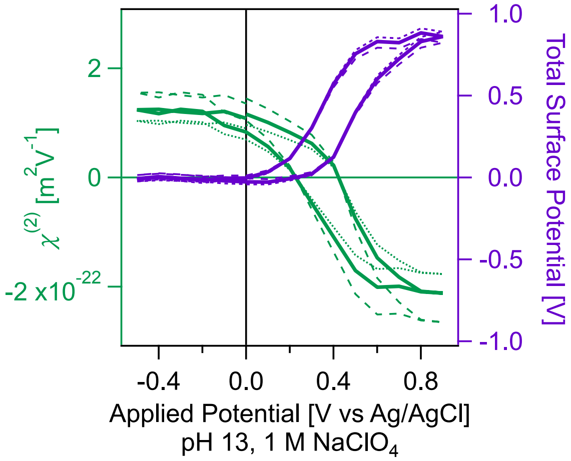


**Fig. S9. Sensitivity analysis.** Second-order nonlinear susceptibility and total surface potential as a function of applied potential computed using a factor of 5 (thick solid lines), 4 (dashed lines), and 6 (dotted lines) in eqn. S4 and eqn. 2 in the main text.

**Supplementary Notes S6.** To assess the utility of vibrational sum frequency generation spectroscopy to count the number of water molecules in the Stern layer, we employed an optical parametric amplifier (LightConversion Orpheus) pumped by an Ytterbium crystal-based laser amplifier (LightConversion Pharos, 10W, 100 kHz, operating with a 10 kHz pulse picker). We used the 1030 nm nanometer residual pump as the upconverter after spectral narrowing by means of an ultranarrow etalon having an optical density of 8 (Edmund Optics Stock# 15-401). Spectra were recorded between 2800 $cm^{-1}$ and 4000 $cm^{-1}$ in 50 $cm^{-1}$ steps using a 300 msec data acquisition time.

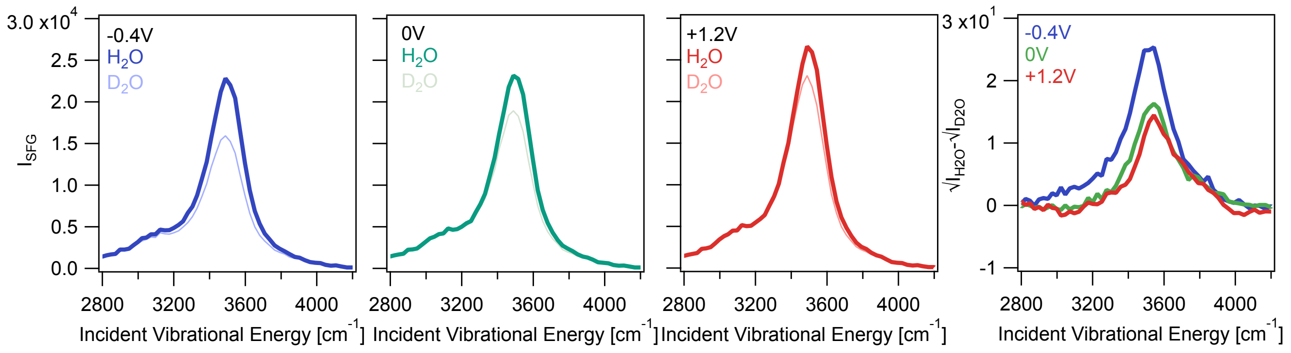


**Fig. S10. Vibrational SFG spectroscopy.** Vibrational sum frequency generation spectra of a 10 nm thin nickel nanolayer on a glass microscope slide recorded in internal reflection at the indicated applied potentials using $D_2O$ and $H_2O$ electrolyte prepared with 0.9M $NaClO_4$ and 0.1M NaOD and NaOH, respectively, and differences of the square rooted SFG intensities recorded using the hydrogenated versus deuterated solutions (rightmost graph).

**Fig. S10** shows the results for applied potentials of -0.4, zero and +1.2 V. The alkaline solutions were prepared using NaOD and $D_2O$ and NaOH and $H_2O$ and adjusted to one M ionic strength with $NaClO_4$. The spectra were recorded in an internal reflection geometry and using 10 nm thin nickel nanolayers. The difference spectra of the square-rooted SFG intensity spectra from the hydrogenated versus deuterated cases show a spectral peak near 3550 wave numbers, comparable to report it SFG Spectra from gold: electrolyte interfaces held under acidic conditions and various potentials (*27*). A quantification how many water molecules point one way or the other is currently hampered by several issues: one is rooted in the convolution of the observed SFG spectral line shape of the $Im(\chi^{(2)}_{total})$ recorded at the detector in a phase-resolved SFG experiment due to the second- and third-order nonlinear optical contributions, the latter of which contains the total surface potential reported in this work. The relevant equation here is the generic expression

$$\chi^{(2)}_{total} = \chi^{(2)}_{surface} + \chi^{(3)}_{water}\,\Phi(0)_{tot} cos(\varphi_{DC}) \cdot e^{i\varphi_{DC}} \qquad \text{eqn. S7}$$

where the DC field phase angle $\varphi_{DC} = \arctan(\Delta k_z\,\lambda_D)$ with $\Delta k_z$ being the wavevector mismatch and $\lambda_D$ being the Debye length in the aqueous solution (*59*, *90-93*). A second issue is the unknown frequency dependence of water's molecular hyperpolarizability in the various hydrogen bonding environments at the interface. While the first problem can be overcome using recently established experimental protocols yielding the Im($\chi^{(2)}_{surface}$) of the interface (*94, 95*), the second problem currently prevents us from turning the Im($\chi^{(2)}_{surface}$) spectra into a net number of Stern layer water molecules pointing up or down in the various relevant interfacial hydrogen bonding environments.

**REFERENCES AND NOTES**


1. Y. Zhang, H. B. de Aguiar, J. T. Hynes, D. Laage, Water structure, dynamics, and sum-frequency generation spectra at electrified graphene interfaces. *J. Phys. Chem. Lett.* **11**, 624–631 (2020).

2. S. Sakong, A. Groß, Water structures on a Pt(111) electrode from ab initio molecular dynamic simulations for a variety of electrochemical conditions. *Phys. Chem. Chem. Phys.* **22**, 10431–10437 (2020).

3. J. Rossmeisl, K. D. Jensen, A. S. Petersen, L. Arnarson, A. Bagger, M. Escudero-Escribano, Realistic cyclic voltammograms from Ab initio simulations in alkaline and acidic electrolytes. *J. Phys. Chem. C* **124**, 20055–20065 (2020).

4. O. M. Magnussen, A. Gross, Toward an atomic-scale understanding of electrochemical interface structure and dynamics. *J. Am. Chem. Soc.* **141**, 4777–4790 (2019).

5. C. Zhang, J. Hutter, M. Sprik, Coupling of surface chemistry and electric double layer at $TiO_2$ electrochemical interfaces. *J. Phys. Chem. Lett.* **10**, 3871–3876 (2019).

6. J. Vatamanu, O. Borodin, Ramifications of water-in-salt interfacial structure at charged electrodes for electrolyte electrochemical stability. *J. Phys. Chem. Lett.* **8**, 4362–4367 (2017).

7. Z. Futera, N. J. English, Water breakup at $Fe_2O_3$−hematite/water interfaces: Influence of external electric fields from nonequilibrium Ab Initio molecular dynamics. *J. Phys. Chem. Lett.* **12**, 6818–6826 (2021).

8. J. L. Bañuelos, E. Borguet, G. E. Brown Jr., R. T. Cygan, J. D. DeYoreo, P. M. Dove, M.-P. Gaigeot, F. M. Geiger, J. M. Gibbs, V. H. Grassian, A. G. Ilgen, Y.-S. Jun, N. Kabengi, L. Katz, J. D. Kubicki, J. Lützenkirchen, C. V. Putnis, R. C. Remsing, K. M. Rosso, G. Rother, M. Sulpizi, M. Villalobos, H. Zhang, Oxide–and silicate–water interfaces and their roles in technology and the environment. *Chem. Rev.* **123**, 6413–6544 (2023).

9. P. Xu, A. D. von Rueden, R. Schimmenti, M. Mavrikakis, J. Suntivich, Optical method for quantifying the potential of zero charge at the platinum-water electrochemical interface. *Nat. Mater.* **22**, 503–510 (2023).

10. S. M. Piontek, M. J. Dellostritto, B. Mandal, T. Marshall, M. L. Klein, E. Borguet, Probing heterogeneous charge distributions at the α-$Al_2O_3$(0001)/$H_2O$ interface. *J. Am. Chem. Soc.* **142**, 12096–12105 (2020).

11. A. Ge, G. Kastlunger, J. Meng, P. Lindgren, J. H. Song, Q. Liu, A. Zaslavsky, T. Lian, A. A. Peterson, On the coupling of electron transfer to proton transfer at electrified interfaces. *J. Am. Chem. Soc.* **142**, 11829–11834 (2020).

12. C.-Y. Li, J.-B. Le, Y.-H. Wang, S. Chen, Z.-L. Yang, J.-F. Li, J. Cheng, Z.-Q. Tian, In situ probing electrified interfacial water structures at atomically flat surfaces. *Nat. Mat.* **18**, 697–701 (2019).

13. S.-J. Shin, D. H. Kim, G. Bae, S. Ringe, H. Choi, H.-K. Lim, C. H. Choi, H. Kim, On the importance of the electric double layer structure in aqueous electrocatalysis. *Nat. Comm.* **13**, 174 (2022).

14. G. Gonella, E. H. G. Backus, Y. Nagata, D. J. Bonthuis, P. Loche, A. Schlaich, R. R. Netz, A. Kühnle, I. T. McCrum, M. T. M. Koper, M. Wolf, B. Winter, G. Meijer, R. K. Campen, M. Bonn, Water at charged interfaces. *Nat. Rev. Chem.* **5**, 466–485 (2021).

15. F. J. Sarabia, P. Sebastián-Pascual, M. T. M. Koper, V. Climent, J. M. Feliuy, Effect of the interfacial water structure on the hydrogen evolution reaction on Pt(111) modified with different nickel hydroxide coverages in alkaline media. *ACS Appl. Mater. Interfaces* **11**, 613–623 (2019).

16. M. R. Nellist, F. A. L. Laskowski, J. Qiu, H. Hajibabaei, K. Sivula, T. W. Hamann, S. W. Boettcher, Potential-sensing electrochemical atomic force microscopy for in operando analysis of water-splitting catalysts and interfaces. *Nat. Energy* **3**, 46–52 (2018).

17. Z. Liang, H. S. Ahn, A. J. Bard, A study of the mechanism of the hydrogen evolution reaction on nickel by surface interrogation scanning electrochemical microscopy. *J. Am. Chem. Soc.* **139**, 4854–4858 (2017).

18. K. Ataka, T. Yotsuyanagi, M. Osawa, Potential-dependent reorientation of water molecules at an electrode/electrolyte interface studied by surface-enhanced infrared absorption spectroscopy. *J. Phys. Chem.* **100**, 10664–10672 (1996).

19. F. Song, L. Bai, A. Moysiadou, S. Lee, C. Hu, L. Liardet, X. Hu, Transition metal oxides as electrocatalysts for the oxygen evolution reaction in alkaline solutions: An application-inspired renaissance. *J. Am. Chem. Soc.* **140**, 7748–7759 (2018).

20. A. J. Bard, L. R. Faulkner, *Electrochemical Methods: Fundamentals and Applications*. (John Wiley and Sons, ed. 2nd, 2000).

21. Y. Wang, T. Seki, X. Liu, X. Yu, C.-C. Yu, K. F. Domke, J. Hunger, M. T. M. Koper, Y. Chen, Y. Nagata, M. Bonn, Direct probe of electrochemical pseudocapacitive pH jump at a graphene electrode. *Angew. Chem. Int. Ed. Engl.* **62**, e202216604 (2023).

22. A. Montenegro, C. Dutta, M. Mammetkuliev, H. Shi, H. Hou, D. Bhattacharyya, B. Zhao, S. B. Cronin, A. V. Benderskii, Asymmetric response of interfacial water to applied electric fields. *Nature* **594**, 62–65 (2021).

23. Y. Tong, F. Lapointe, M. Thaemer, M. Wolf, R. K. Campen, Experimentally probing hydrophobic water at the gold electrode / aqueous interface. *Angew. Chemie Int. Ed. Engl.* **56**, 4211–4214 (2017).

24. N. Garcia Rey, D. D. Dlott, Studies of electrochemical interfaces by broadband sum frequency generation. *J. Electroanal. Chem.* **800**, 114–125 (2017).

25. S. Nihonyanagi, T. Tahara, Direct evidence for orientational flip-flop of water molecules at charged interfaces: A heterodyne-detected vibrational sum frequency generation study. *J. Chem. Phys.* **130**, 204704 (2009).

26. S. Baldelli, Probing electric fields at the ionic liquid-electrode interface using sum frequency generation spectroscopy and electrochemistry. *J. Phys. Chem. B* **109**, 13049–13051 (2005).

27. S. Nihongyanagi, S. Ye, K. Uosaki, L. Dreesen, C. Humbert, P. A. Thiry, A. Peremans, Potential-dependent structure of the interfacial water on the gold electrode. *Surf. Sci.* **573**, 11–16 (2004).

28. B. M. Biwer, M. J. Pellin, M. W. Schauer, D. M. Gruen, Electrochemical and second harmonic generation investigation of nickel corrosion in 0.1 M NaOH. *Surf. Interf. Anal.* **14**, 635–646 (1989).

29. G. Nagy, D. Roy, Surface charge dependence of second harmonic generation from a Ni electrode. *Chem. Phys. Lett.* **214**, 197–202 (1993).

30. C. H. Lee, R. K. Chang, N. Bloembergen, Nonlinear electroreflectance in silicon and silver. *Phys. Rev. Lett.* **18**, 167–170 (1967).

31. G. L. Richmond, Surface second harmonic generation from sulfate ions adsorbed on silver electrodes. *Chem. Phys. Lett.* **106**, 26–29 (1984).

32. R. M. Corn, M. Romagnoli, M. D. Levenson, M. R. Philpott, The potential dependence of surface plasmon-enhanced second-harmonic generation at thin film silver electrodes. *Chem. Phys. Lett.* **106**, 30–35 (1984).

33. M. S. A. Akbari, R. Bagheri, Z. Song, M. M. Najafpour, Oxygen-evolution reaction by nickel/nickel oxide interface in the presence of ferrate (VI). *Sci. Rep.* **10**, 8757 (2020).

34. E. Ma, P. E. Ohno, K. Kim, Y. Liu, E. H. Lozier, T. F. Miller III, H.-F. Wang, F. M. Geiger, A new imaginary term in the 2nd order nonlinear susceptibility from charged interfaces. *J. Phys. Chem. Lett.* **12**, 5649–5659 (2021).

35. L. Dalstein, K.-Y. Chiang, Y.-C. Wen, Direct quantification of water surface charge by phase-sensitive second harmonic spectroscopy. *J. Phys. Chem. Lett.* **10**, 5200–5205 (2019).

36. P. Guyot-Sionnest, A. Tadjeddine, A. Liebsch, Electronic distributions and nonlinear optical response at the metal-electrolyte interface. *Phys. Rev. Lett.* **64**, 1678–1681 (1990).

37. C. Lütgebaucks, G. Gonella, S. Roke, Optical label-free and model-free probe of the surface potential of nanoscale and microscopic objects in aqueous solution. *Phys. Rev. B* **94**, 195410 (2016).

38. Y.-C. Wen, S. Zha, X. Liu, S. Yang, P. Guo, G. Shi, H. Fang, Y. R. Shen, C. Tian, Unveiling microscopic structures of charged water interfaces by surface-specific vibrational spectroscopy. *Phys. Rev. Lett.* **116**, 016101 (2016).

39. T. Mahmood, M. T. Saddique, A. Naeem, P. Westerhoff, S. Mustafa, A. Alum, Comparison of different methods for the point of zero charge determination of NiO. *Ind. Eng. Chem. Res.* **50**, 10017–10023 (2011).

40. M. Kosmulski, The pH dependent surface charging and points of zero charge. VI. Update. *J. Colloid Interface Sci.* **426**, 209–212 (2014).

41. P. H. Tewari, A. B. Campbell, Temperature dependenceof pointof zero chargeof cobalt and nickel oxidesand hydroxides. *J. Coll. Int. Sci.* **55**, 531–539 (1976).

42. A. L. Olson, A. O. Alghamdi, F. M. Geiger, NaCl, $MgCl_2$, and $AlCl_3$ surface coverages on fused silica and adsorption free energies at pH 4 from nonlinear optics. *J. Phys. Chem. A* **128**, 2162–2168 (2024).

43. L. Bousse, Single electrode potentials related to flat-band voltage measurements on EOS and MOS structures. *J. Chem. Phys.* **76**, 5128–5133 (1982).

44. L. Bousse, N. F. De Rooij, P. Bergveld, Operation of chemically sensitive field-effect sensors as a function of the insulator-electrolyte interface. *IEEE Trans. Electron Devices* **30**, 1263–1270 (1983).

45. J. L. Diot, J. Joseph, J. R. Martin, P. Clechet, pH dependence of the Si/$SiO_2$ interface state density for EOS systems: Quasi-static and AC conductance methods. *J. Electroanal. Chem.* **193**, 75–88 (1985).

46. M. A. Brown, Z. Abbas, A. Kleibert, R. G. Green, A. Goel, S. May, T. M. Squires, Determination of surface potential and electrical double-layer structure at the aqueous electrolyte-nanoparticle interface. *Phys. Rev. X* **6**, 011007 (2016).

47. M. A. Brown, G. V. Bossa, S. May, Emergence of a stern layer from the incorporation of hydration interactions into the Gouy–Chapman model of the electrical double layer. *Langmuir* **31**, 11477–11483 (2015).

48. M. A. Brown, A. Goel, Z. Abbas, Effect of electrolyte concentration on the stern layer thickness at a charged interface. *Angew. Chem. Int. Ed. Engl.* **55**, 3790–3794 (2016).

49. A. V. Gubskaya, P. G. Kusalik, The multipole polarizabilities and hyperpolarizabilities of the water molecule in liquid state: An ab initio study. *Mol. Phys.* **99**, 1107–1120 (2001).

50. L. Fumagalli, A. Esfandiar, R. Fabregas, S. Hu, P. Ares, A. Janardanan, Q. Yang, B. Radha, T. Taniguchi, K. Watanabe, G. Gomila, K. S. Novoselov, A. K. Geim, Anomalously low dielectric constant of confined water. *Science* **360**, 1339–1342 (2018).

51. A. Codello, Ising Model and Phase Transitions (2013); https://universalitylectures.wordpress.com.

52. V. F. Petrenko, R. W. Whitworth, *Physics of Ice*. (Oxford Univ. Press, 1999).

53. E. J. Horn, B. R. Rosen, P. S. Baran, Synthetic organic electrochemistry: An enabling and innately sustainable method. *ACS Centr. Sci.* **2**, 302–308 (2016).

54. M. D. Boamah, E. H. Lozier, J. Kim, P. E. Ohno, C. E. Walker, T. F. I. Miller, F. M. Geiger, Energy conversion via metal nanolayers. *Proc. Natl. Acad. Sci. U.S.A.* **116**, 16210–16215 (2019).

55. R. R. Rao, S. Corby, A. Bucci, M. Garcia-Tecedor, C. A. Mesa, J. Rossmeisl, S. Gimenez, J. Lloret-Fillo, I. E. L. Stephens, J. R. Durrant, Spectroelectrochemical analysis of the water oxidation mechanism on doped nickel oxides. *J. Am. Chem. Sco.* **144**, 7622–7633 (2022).

56. M. D. Boamah, D. Isheim, F. M. Geiger, Dendritic oxide growth in zerovalent iron nanofilms revealed by atom probe tomography. *J. Phys. Chem. C* **122**, 28225–28232 (2018).

57. P. A. Covert, D. K. Hore, Assessing the gold standard: The complex vibrational nonlinear susceptibility of metals. *J. Phys. Chem. C* **119**, 271–276 (2014).

58. K. Kemnitz, K. Bhattacharyya, J. M. Hicks, G. R. Pinto, K. B. Eisenthal, T. F. Heinz, The phase of 2nd-harmonic light generated at an interface and its relation to absolute molecular-orientation. *Chem. Phys. Lett.* **131**, 285–290 (1986).

59. P. E. Ohno, S. A. Saslow, H.-F. Wang, F. M. Geiger, K. B. Eisenthal, Phase-referenced nonlinear spectroscopy of the α-quartz/water interface. *Nat. Commun.* **7**, 13587 (2016).

60. C. Wang, Second-harmonic generation of light at the boundary of an isotropic medium. *Phys. Rev.* **178**, 1457–1461 (1969).

61. T. F. Heinz, C. K. Chen, D. Ricard, Y. R. Shen, Optical 2nd-harmonic generation from a monolayer of centrosymmetric molecules adsorbed on silver. *Chem. Phys. Lett.* **83**, 180–182 (1981).

62. C. K. Chen, T. F. Heinz, D. Ricard, Y. R. Shen, Surface-enhanced second-harmonic generation and Raman scattering. *Phys. Rev. B* **27**, 1965–1979 (1983).

63. H. M. Rojhantalab, G. L. Richmond, Interfacial studies of sliver-aqueous electrolytes by optical second harmonic and differential capacitance. *J. Opt. Soc. Am. A Opt. Image Sci. Vis.* **3**, P52–P53 (1986).

64. G. L. Richmond, Characterization of the silver-aqueous electrolyte interface by optical second harmonic generation. *Langmuir* **2**, 132–139 (1986).

65. G. L. Richmond, Adsorption of ions on smooth and roughened silver surfaces: A comparative study by optical second harmonic generation. *Chem. Phys. Lett.* **113**, 359–363 (1985).

66. G. L. Richmond, H. M. Rojhantalab, J. M. Robinson, V. L. Shannon, Experiments on optical second-harmonic generation as a surface probe of electrodes. *J. Opt. Soc. Am. B Opt. Phys.* **4**, 228–236 (1987).

67. G. L. Richmond, In situ characterization of solid liquid interfaces by optical 2nd harmonic-generation. *J. Electrochem. Soc.* **134**, C111–C111 (1987).

68. J. M. Lantz, R. M. Corn, Time-resolved optical second harmonic generation measurements of picosecond band flattening processes at single crystal Ti02 electrodes. *J. Phys. Chem.* **98**, 9387–9390 (1994).

69. P. Guyot-Sionnest, A. Tadjeddine, Study of Ag(111) and Au(111) Electrodes by Optical Second-harmonic Generation. *J. Chem. Phys.* **92**, 734–738 (1990).

70. S. Ong, X. Zhao, K. B. Eisenthal, Polarization of water molecules at a charged interface; second harmonic studies of the silica/water interface. *Chem. Phys. Lett.* **191**, 327–335 (1992).

71. G. L. Richmond, J. M. Robinson, V. L. Shannon, Second harmonic generation studies of interfacial structure and dynamics. *Prog. Surf. Sci.* **28**, 1–70 (1988).

72. R. M. Corn, D. A. Higgins, Optical second harmonic generation as a probe of surface chemistry. *Chem. Rev.* **94**, 107 (1994).

73. K. B. Eisenthal, Liquid interfaces probed by second-harmonic and sum-frequency spectroscopy. *Chem. Rev.* **96**, 1343–1360 (1996).

74. K. B. Eisenthal, Second harmonic spectroscopy of aqueous nano- and microparticle interfaces. *Chem. Rev.* **106**, 1462–1477 (2006).

75. S. Baldelli, N. Markovic, P. Ross, Y. R. Shen, G. Somorjai, Sum frequency generation of CO on (111) and polycrystalline platinum electrode surfaces: Evidence for SFG invisible surface CO. *J. Phys. Chem. B* **103**, 8920–8925 (1999).

76. S. Baldelli, G. Mailhot, P. Ross, Y. R. Shen, G. A. Somarjai, Potential dependent orientation of acetonitrile on platinum (111) electrode surface studied by sum frequency generation. *J. Phys. Chem. B.* **105**, 654–662 (2001).

77. S. K. Shaw, A. Lagutchev, D. D. Dlott, A. A. Gewirth, Electrochemically driven reorientation of three ionic states of p-Aminobenzoic Acid on Ag(111). *J. Phys. Chem. C* **113**, 2417–2424 (2009).

78. S. K. Shaw, A. Lagutchev, D. D. Dlott, A. A. Gewirth, Sum-frequency spectroscopy of molecular adsorbates on low-index Ag surfaces: Effects of azimuthal rotation. *Anal. Chem.* **81**, 1154–1161 (2009).

79. N. Garcia Rey, D. D. Dlott, Structural transition in an ionic liquid controls $CO_2$ electrochemical reduction. *J. Phys. Chem. C* **119**, 20892–20899 (2015).

80. Y. Tong, K. Cai, M. Wolf, R. K. Campen, Probing the electrooxidation of weakly adsorbed formic acid on Pt(1 0 0). *Catal. Today.* **260**, 66–71 (2016).

81. I. Nahalka, G. Zwaschka, R. K. Campen, A. Marchioro, S. Roke, Mapping electrochemical heterogeneity at gold surfaces: A second harmonic imaging study. *J. Phys. Chem. C* **124**, 20021–20034 (2020).

82. G. Zwaschka, I. Nahalka, A. Marchioro, Y. Tong, S. Roke, R. K. Campen, Imaging the heterogeneity of the oxygen evolution reaction on gold electrodes operando: Activity is highly local. *ACS Catal.* **10**, 6084–6093 (2020).

83. H. Wang, Q. Xu, Z. Liu, Y. Tang, G. H. Wei, Y. R. Shen, W.-T. Liu, Gate-controlled sum-frequency vibrational spectroscopy for probing charged oxide/water interfaces. *J. Phys. Chem. Lett.* **10**, 5943–5948 (2019).

84. P. Xu, A. Huang, J. Suntivich, Phase-sensitive second-harmonic generation of electrochemical interfaces. *J. Phys. Chem. Lett.* **11**, 8216–8221 (2020).

85. J. Y. Huang, A. Lewis, Determination of the absolute orientation of the retinylidene chromophore in purple membrane by a second-harmonic interference technique. *Biophys. J.* **55**, 835–842 (1989).

86. R. Stolle, G. Marowsky, E. Schwarzberg, G. Berkovic, Phase measurements in nonlinear optics. *Appl. Phys. B* **63**, 491–498 (1996).

87. E. Ma, F. M. Geiger, Divalent ion specific outcomes on stern layer structure and total surface potential at the silica:water interface. *J. Phys. Chem. A* **125**, 10079–10088 (2021).

88. X. Wei, S. C. Hong, A. I. Lvovsky, H. Held, Y. R. Shen, Evaluation of surface vs bulk contributions in sum-frequency vibrational spectroscopy using reflection and transmission geometries. *J. Phys. Chem. B* **104**, 3349–3354 (2000).

89. J. F. D. Liljeblad, E. Tyrode, Vibrational sum frequency spectroscopy studies at solid/liquid interfaces: Influence of the experimental geometry in the spectral shape and enhancement. *J. Phys. Chem. C* **116**, 22893–22903 (2012).

90. P. E. Ohno, H.-F. Wang, F. M. Geiger, Second-order spectral lineshapes from charged interfaces. *Nat. Commun.* **8**, 1032 (2017).

91. S. K. Reddy, R. Thiraus, B. A. W. Rudd, L. Lin, T. Adel, T. Joutsuka, F. M. Geiger, H. C. Allen, A. Morita, F. Paesani, Bulk contributions modulate the sum-frequency generation spectra of water on model sea-spray aerosols. *Chem* **4**, 1629–1644 (2018).

92. P. E. Ohno, H. F. Wang, F. Paesani, J. L. Skinner, F. M. Geiger, Second-order vibrational lineshapes from the air/water interface. *J. Phys. Chem. A* **122**, 4457–4464 (2018).

93. P. E. Ohno, H. Chang, A. P. Spencer, Y. Liu, M. D. Boamah, H.-F. Wang, F. M. Geiger, Correction to beyond the gouy-chapman model with heterodyne-detected second harmonic generation. *J. Phys. Chem. Lett.* **10**, 5364 (2019).

94. B. Rehl, E. Ma, S. Parshotam, E. L. DeWalt-Kerian, T. Liu, F. M. Geiger, J. M. Gibbs, water structure in the electrical double layer and the contributions to the total interfacial potential at different surface charge densities. *J. Am. Chem. Soc.* **144**, 16338–16349 (2022).

95. F. Wei, S.-H. Urashima, S. Nihongyanagi, T. Tahara, Elucidation of the pH-dependent electric double layer structure at the silica/water interface using heterodyne-detected vibrational sum frequency generation spectroscopy. *J. Am. Chem. Soc.* **145**, 8833–8846 (2023).
